# Effects of different epidural initiation volumes on postoperative analgesia in cesarean section

**DOI:** 10.3906/sag-1905-44

**Published:** 2020-12-17

**Authors:** Osman KAÇMAZ, Nurçin GÜLHAŞ, Gülay ERDOĞAN KAYHAN*, Mahmut DURMUŞ

**Affiliations:** 1 Department of Anesthesiology and Reanimation, Malatya Traning and Research Hospital, Malatya Turkey; 2 Department of Anesthesiology and Reanimation, Faculty of Medicine, İnönü University, Malatya Turkey; 3 Department of Anesthesiology and Reanimation, Faculty of Medicine, Eskişehir Osmangazi University, Eskişehir Turkey; 4 Department of Anesthesiology and Reanimation, Faculty of Medicine, İnönü University, Malatya Turkey

**Keywords:** Cesarean section, combined spinal epidural anesthesia, patient-controlled epidural analgesia, postoperative pain

## Abstract

**Background/aim:**

The aim of this study was to compare the effects of different epidural initiation volumes on postoperative pain scores, analgesic requirements, and side effects in pregnant women administered patient-controlled epidural analgesia (PCEA) for postoperative pain after cesarean sections.

**Materials and methods:**

Eighty-one pregnant women, aged 18–45 years, were included in this randomized, double-blind study. Combined spinal epidural anesthesia was administered for each cesarean section. The patients were divided into 3 groups and different volumes (20 mL, 10 mL, and 5 mL) of the study drug (0.0625% bupivacaine plus 2 μg/mL of fentanyl) were administered 90 min after the spinal block via epidural catheter. The visual analogue scale (VAS) scores at rest and during movement, first PCEA dose time, number of PCEA doses required per hour, total analgesic consumed, and side effects were recorded postoperatively.

**Results:**

There were no statistically significant differences among the groups in terms of the VAS rest and VAS movement scores. The times to the first analgesic dose requirement were longer in Group 10 and Group 20 than in Group 5. The analgesic requirement during the first 2 h was lower in Group 20 than in the other groups.

**Conclusions:**

The PCEA initiations with different volumes provided similar pain scores. However, the 20 mL volume resulted in a lower analgesic dose requirement during the early postoperative period, and it also delayed the requirement for analgesia.

## 1. Introduction

Postoperative pain is a major source of concern among patients undergoing cesarean sections because pain intensity after a cesarean section can be high. A strong interaction between the mother and infant during the early postnatal period has psychological importance with regard to the optimal development of the infant. Adequate pain relief contributes to the development of the mother-infant bond, early ambulation, early hospital discharge, and a decrease in deep vein thrombosis risk [1–4]. Therefore, it is important to provide effective and adequate pain relief.

Nowadays, patient-controlled analgesia (PCA) devices are widely used for epidural or intravenous postoperative analgesia. However, the intravenous administration of higher doses of medications increases the incidence of side effects. With epidural analgesia, fewer systemic effects are observed, while longer and more reliable analgesia is provided at lower drug dosages [5]. Epidural analgesia can be initiated at a range of volumes, but there is inadequate data about the exact volumes and PCA protocols after a cesarean section [6,7]. In some previous studies, higher volumes of epidural analgesia provided better pain scores when compared to lower volumes [8,9]. Conversely, some studies showed that the delivery of small volumes (5 mL) via the epidural route was also sufficient [7].

The primary aim of this randomized, double-blind study was to compare the effects of different epidural initiation volumes (5, 10, and 20 mL) on the postoperative pain scores of patients who were administered patient-controlled epidural analgesia (PCEA) to treat postoperative pain after a cesarean section. The secondary aim was to compare the first analgesic dose demand times, number of analgesic doses, morphine requirements, and side effects.

## 2. Materials and methods

This study was conducted after obtaining Ethics Committee approval from the İnönü University Medical Faculty (2015/171) and informed consent from all of the participants. Pregnant women with American Society of Anesthesiologists class II physical status, aged 18–45 years, and underwent cesarean sections at the Turgut Özal Medical Center Hospital of İnönü University were included in the study. Patients with multiple pregnancies, diabetes mellitus, hypertension requiring treatment, bleeding diathesis, anticoagulant use, and histories of severe cardiac, neurological and pulmonary diseases were excluded from the study. In addition, pregnant women with bupivacaine and fentanyl allergies and difficulty in understanding the use of the PCA device and the pain scoring system were not included in the study. Before surgery, each patient was informed about the PCA device and the visual analogue scale (VAS).

After each unpremedicated patient was taken to the operating room, we began an infusion of 4 mL/kg of Ringer’s lactate solution per hour via establishing vascular access. Standardized monitoring was achieved via electrocardiography, noninvasive blood pressure (NIBP), peripheral oxygen saturation and heart rate (HR) values. The baseline systolic arterial pressure (SAP) and mean arterial pressure (MAP) values were obtained by taking the average of 3 measured NIBP values at 2 min intervals.

For the combined spinal-epidural anesthesia (CSE), the epidural space was accessed from the L3–4 or L4–5 levels using an 18 G Tuohy needle (18 G x 90 mm; Egemen International, İzmir, Turkey) with the loss of resistance method while the patient was in a sitting position. After the identification of the epidural space, a spinal block was performed using 10 mg of heavy bupivacaine with a 27 G Quincke spinal needle (27 G x 135 mm; Egemen International) using a needle-through-needle technique. Then, a soft tip radiopaque 20 G catheter (20 G x 100 cm; Egemen International) with a lateral hole was advanced 4 cm into the epidural space and fixed. The surgery began when the sensory block reached the T4–6 level based on a pinprick test of the middle clavicular line using a needle tip.

The study drugs were administered through the epidural catheter 90 min after the block was performed. The patients were randomly divided into 3 groups using the envelope method. A 20 mL dose of 0.0625% bupivacaine plus 2 μg/mL of a fentanyl solution was administered to the patients in Group 20 (n = 27). A 10 mL dose of 0.0625% bupivacaine plus 2 μg/mL of a fentanyl solution was administered to the patients in Group 10 (n = 27). A 5 mL dose of 0.0625% bupivacaine plus 2 μg/mL of a fentanyl solution was administered to the patients in Group 5 (n = 27). The study drug was administered through the epidural catheter in 5 mL divided doses, and the first 5 mL dose was accepted as the test dose. A nurse anesthesiologist prepared the study drugs and covered them with a black sheath so that the doses were not recognized. The study drugs were administered by a member of the research team. The patient, surgical team, and anesthesiologist who collected the postoperative data were all blinded to the study drugs.

In each group, the 0.0625% bupivacaine plus 2 μg/mL of a fentanyl solution was started through the epidural catheter using the PCA device as follows: no baseline infusion, a bolus dose of 5 mL, and a lockout interval of 15 min. In addition, a 50 μg/kg dose of morphine in 10 mL of saline was administered through the epidural catheter to the patients who needed to use the PCEA more than 4 times per h. A decrease of 30%, according to the baseline systolic blood pressure, was considered to be hypotension, and this was planned to treat with 10 mg of ephedrine.

Each patient was followed up for at least 30 min in the postoperative care unit, and then sent to the Gynecology and Obstetrics Service after her vital signs were stabilized. The VAS scores at rest and during movement (using the pain marking method on a 10 cm ruler with 0 at the beginning and 10 at the end), the first analgesic demand time (first PCEA demand time), the number of PCEA requirements per hour (number of PCA requests), the morphine requirements, and hemodynamic data of the patients in the recovery room at 2, 4, 6, and 12 h postoperatively were recorded. Nausea and vomiting, itching, motor block, and hypotension, as well as the ephedrine amounts, were also recorded at the same follow-up time periods. The motor block resolution time was assessed using the modified Bromage scale (0 = no paralysis, and the patient can bring her foot and knee to full flexion: 1 = the patient can only move only her knee and leg, she cannot lift her leg straight; 2 = the patient cannot bend her knee, she can only move the leg; 3 = the patient cannot move her ankle or thumb, there is full paralysis).

### 2.1. Statistical analysis

For the power analysis, it was calculated that at least 24 cases should be included in each group in order to detect an average change of 23 mm in the VAS score, when α = 0.05 and 1 - β (power) = 0.80. When taking into consideration study withdrawals and protocol violations, 27 cases were included in each group in this study. We used the Statistical Package for the Social Sciences (version 17.0 for Windows; SPSS Inc., Chicago, IL, USA) for the statistical analysis of our research data. The mean ± standard deviation was used to define the quantitative variable data, and the number (n) and percentage (%) were used for the qualitative variable data. The quantitative variable data were tested using the Shapiro–Wilk normality test. According to the test results, the comparisons between more than 2 groups were performed using a one-way analysis of variance and the Kruskal–Wallis analysis of variance. The comparison of 2 groups was performed using the Mann–Whitney U test. The Wilcoxon test was used for the analyses of the intragroup variables. The evaluation of the qualitative variables was conducted using the Pearson chi-square analysis. A P value of < 0.05 was considered to be statistically significant.

## 3. Results

**Figure 1:**
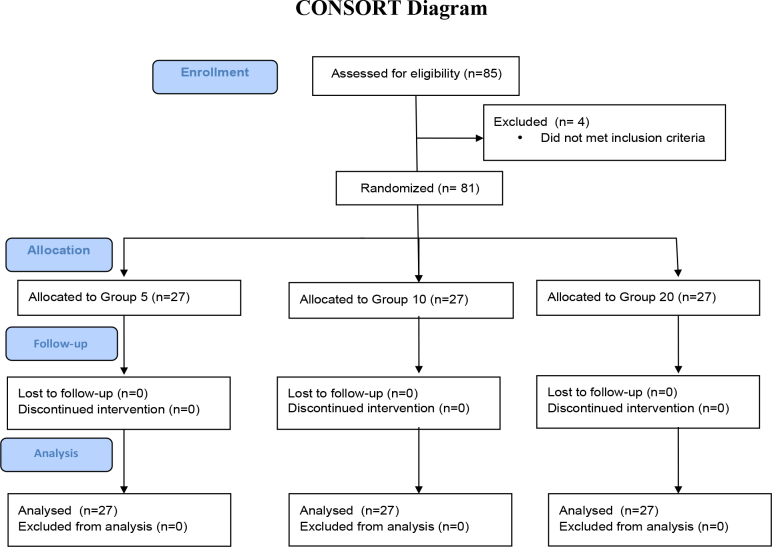
Flow diagram of study participants

A total of 85 patients were assessed for eligibility (Figure 1). Of them, 81 were enrolled in the study. The patients’ demographic data were similar among the groups (Table 1).

**Table 1:** Demographic data of patients

	Group 5 (n = 27)	Group 10 (n = 27)	Group 20 (n = 27)
Age (years)	30 ± 4.5	30 ± 5.4	30.8 ± 4.9
Height (cm)	162.1 ± 6.2	162.2 ± 5	162.2 ± 5.6
Weight (kg)	78.1 ± 13.7	79.2 ± 10.7	77.6 ± 14.7

P > 0.05

When the baseline and postoperative values of HR, SAP, and MAP were compared, it was determined that there were no statistically significant differences among the groups (P > 0.05) (Table 2).

**Table:** Hemodynamic data.

		T0	T1	T2	T3	T4	T5
HR(beat/min)	Group 5 (n = 27)	99.1 ± 15.1	100.2 ± 5.1	99.6 ± 4.7	99.1 ± 6.3	99.1 ± 2.8	99 ± 1.6
	Group 10 (n = 27)	99 ± 13.7	99.4 ± 8.1	99.1 ± 7.5	99 ± 8.4	99 ± 7.9	98 ± 1.1
	Group 20 (n = 27)	95.6 ± 13	98.2 ± 1.4	98 ± 1.2	98.1 ± 4.6	98.1 ± 2.5	97 ± 3.5
SAP(mmHg)	Group 5 (n = 27)	126.2 ± 18.9	130 ± 3.2	130 ± 8.8	130 ± 7.3	130 ± 6.5	128 ± 5.2
	Group 10 (n = 27)	126.6 ± 14	128.4 ± 1.2	128.8 ± 5.4	128 ± 2.4	127.9 ± 3.6	127 ± 2.8
	Group 20 (n = 27)	132 ± 17.3	126.5 ± 9.6	127 ± 4.2	127 ± 1.1	126.7 ± 4.5	126 ± 7.6
MAP (mmHg)	Group 5 (n = 27)	96.8 ± 10.1	97 ± 9.8	97.2 ± 8.7	97 ± 5.3	97 ± 2.2	96 ± 1.3
	Group 10 (n = 27)	92.3 ± 11.1	96.5 ± 8.6	96.6 ± 9.1	96 ± 4.8	96 ± 3.1	95 ± 2.1
	Group 20 (n = 27)	95.1 ± 12.5	95.3 ± 6.1	96 ± 4.6	96 ± 1.2	95.9 ± 1.2	95 ± 3.6

T0: baseline values before operation; T1: values before epidural initiation volume injection; T2: values 2 h after epidural initiation volume injection; T3: values 4 h after epidural initiation volume injection; T4: values 6 h after epidural initiation volume injection; T5: values 12 h after epidural initiation volume injection (P > 0.05).

**Figure 2:**
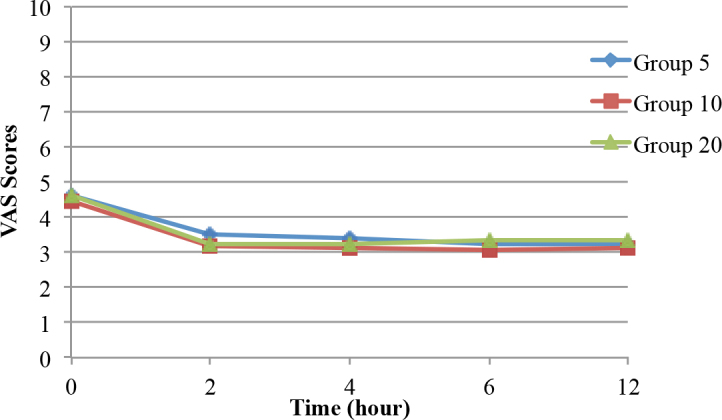
Change of VAS rest scores over time.

**Figure 3:**
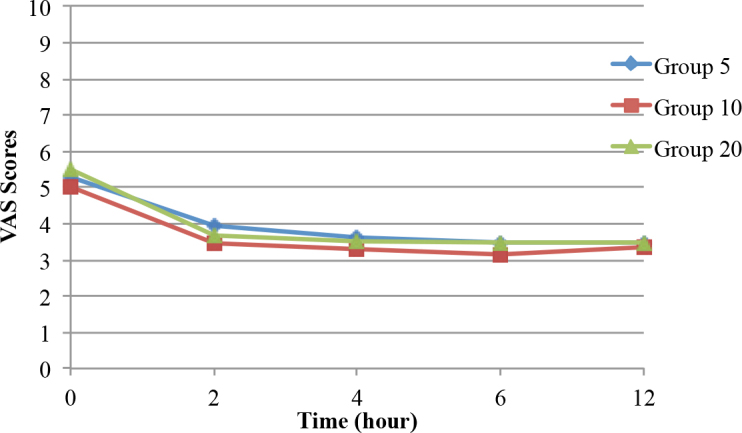
Change of VAS movement scores over time.

**Figure 4:**
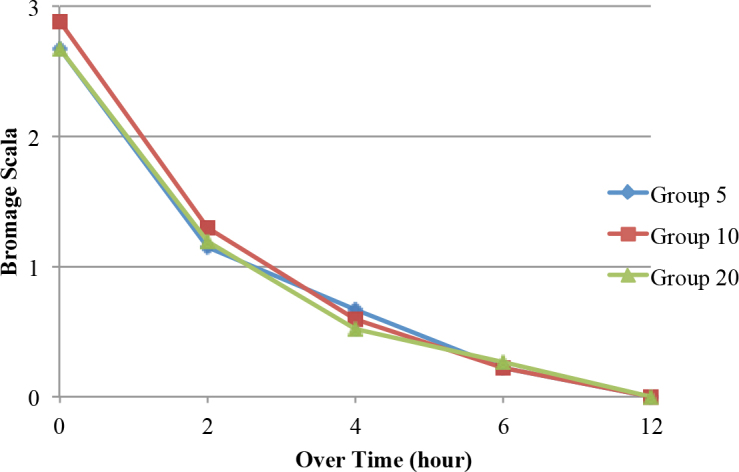
Change of motor block over time.

There were no statistically significant differences among the groups in terms of the VAS rest and VAS movement scores. In the intragroup comparisons, the VAS rest and VAS movement scores were significantly lower than the baseline values in all 3 groups (P < 0.05) (Figures 2 and 3). In addition, there were no statistically significant differences in terms of the motor blocks during all of the follow-up periods among the groups (P > 0.05) (Figure 4). The first analgesic dose demand times (first PCA times) were 44.89 ± 26.73 min in Group 5, 85.93 ± 64.51 min in Group 10, and 97.96 ± 66.17 min in Group 20. The first analgesic dose demand times in Group 10 and Group 20 were significantly longer than that in Group 5 (P = 0.004).

The analgesic dose requirements (number of PCA requests) during the first 2 h were 3.30 ± 1.4 in Group 5, 2.07 ± 1.73 in Group 10, and 1.41 ± 1.57 in Group 20. In Group 20, the number of PCA requests was significantly lower than those in the other groups, but only during the first 2 h (P < 0.001).

There were no significant differences among the groups in terms of the total analgesics and the total ephedrine consumed (P > 0.05) (Table 3). No morphine was required by any of the patients in the groups.

**Table 3:** Total consumed analgesic and ephedrine amounts.

	Group 5 (n = 27)	Group 10 (n = 27)	Group 20 (n = 27)
Total consumed analgesic (mL)	82.4 ± 25	71.4 ± 33.9	77.4 ± 29.5
Ephedrine requirement, n (%)	0	2 (7.4%)	0

P > 0.05

The itching score values of the groups at the 2nd, 4th, 6th, and 12th h were compared with baseline bolus values. There were significant differences in the itching scores at the 4th, 6th, and 12th h when compared to the itching score during the bolus period in Group 5 (P < 0.05). There were significant differences in the itching scores at the 6th and 12th h when compared to the itching score during the bolus period in Group 10 (P < 0.05). However, there were no statistically significant differences found in Group 20 (P > 0.05). In the between-group comparisons, the itching score in Group 10 at 6th h was significantly higher than those in the other 2 groups (P > 0.05). The itching incidences during all of the follow-up periods in the groups are shown in Table 4.

**Table 4:** Incidence of itching and nausea.

	Group 5, n (%)	Group 10, n (%)	Group 20, n (%)	P value
Itching	8 (29.6)	12 (44.4)	3 (11.1)	0.02
Nausea	6 (22.2)	5 (18.5)	6 (22.2)	0.92

The patients were compared to each other within the groups and between the groups in terms of the nausea scores and incidences. In the within-group comparisons, there were no statistically significant differences in the nausea scores of the bolus or at the 2nd, 4th, 6th, or 12th h postoperatively (P > 0.05). In the between-group comparisons, the groups were similar in terms of nausea scores (P < 0.05). Nausea incidences are provided in Table 4.

## 4. Discussion

In our study, the administrations of 5 mL, 10 mL, and 20 mL for the epidural initiation volumes provided similar pain scores and motor block resolution times in the patients who were given PCEA for postoperative pain after cesarean sections. However, when we used the 20 mL volume to initiate the epidural analgesia, we determined that time to the first analgesic requirement was longer and the analgesic requirement during the first 2 h was less than initial volumes of 5 mL and 10 mL without any hemodynamic disturbance.

Epidural anesthesia and analgesia are commonly used for obstetric anesthesia. Epidural anesthesia is safe for use in cesarean sections, even in patients with cardiac diseases and severe pulmonary hypertension [4,7,10–16]. Several methods, including wound infiltration with a local anesthetic and magnesium sulphate, wound infiltration with tramadol and levobupivacaine, a magnesium sulphate infusion for preemptive analgesia, a transverse abdominal plane block (TAPB) performed by adding dexamethasone to levobupivacaine, infiltration by placing a continuous catheter for a TAPB or the wound area, the administration of intrathecal and intravenous betamethasone, and the addition of dexmedetomidine to increase the efficacy of epidural anesthesia and analgesia have all been used to treat pain after a cesarean section [17–23]. We administered epidural analgesia to our patients in order to avoid the side effects of systemically administered opioids, such as nausea and vomiting, urinary retention, pruritus, and respiratory depression, and to reduce the postoperative antiinflammatory response [24–25].

CSE anesthesia was preferred in our study because it combines the advantages of spinal anesthesia (rapid onset, minimal toxic effects, high efficacy) and epidural anesthesia (lengthening the duration of anesthesia), partially reduces their disadvantages, and allows for postoperative analgesia [15,25–28]. However, we waited 90 min after the spinal block so that the effects of the spinal bupivacaine had passed before performing our postoperative objective analyses in all 3 groups. At the end of this period, the epidural catheter was activated.

Pure local anesthetic use for epidural analgesia is not a common method due to inadequate pain relief and prolonged motor block. The addition of opioids as an adjunct to postcesarean pain treatment improves the quality of the block. In our study, we added fentanyl to the bupivacaine as in the literature [14,29,30]. Moreover, we administered PCEA to our patients because it was found that epidural bolus in conjunction with the PCEA regimen was of greater benefit to the parturient and fetus [7,31].

In cesarean sections, analgesic dose requirements occur more frequently in the patients during the first 12 postoperative hours [1]. Therefore, we set our observation periods within the first 12 h. The reason why there was no difference in the amount of the total analgesic consumed in our study may have been that we used the test volumes (5, 10, or 20 mL) only when initiating the analgesia. If we had set the bolus dose higher on the PCEA device, we may have been able to detect a difference.

It has been previously reported that the most important factors for determining block quality in epidural anesthesia and analgesia are the volume and distribution surface [32,33]. In a study conducted by Bernard et al., successful results were achieved using 20 mL. Unlike our study, they carried out a labor analgesia study [34]. In another study about labor analgesia, conducted by Song et al., the epidural analgesic success rate was determined by the volume given through the epidural [35].

In a study conducted by Christiaens et al., better analgesia quality was provided in the patients with a 20 mL diluted volume of anesthetic when compared to the patients with a 10 mL volume; however, there was a difference between the groups in terms of motor block and VAS scores in that study [8]. The reason why we did not detect differences in terms of motor block and VAS scores may have been the lower concentrations of local anesthetic concentrations in our study. Low concentrations of local anesthetics are known to cause less of a motor block [32]. In the study conducted by Christiaens et al., it was reported that the analgesia was continued during the first 2 h in the 20 mL group, which was similar to our study [8].

In a study conducted by Rabinovitch et al., it was shown that the pain was better controlled in the patients who received high-volume epidurals for radicular leg and back pain, and there was a positive correlation between the volume and the pain relief [36]. Similarly, we observed better pain control in the patients in which we initiated the epidural analgesia at high volumes in our study.

In a study conducted by Cohen et al., PCEA was used for postcesarean pain, similar to our study, but they added a 10 mL/s basal infusion and used epinephrine in addition to the local anesthetic and fentanyl [29].

Sng et al. determined that there were no differences between a group with a 5 mL infusion and a group without an infusion [7]. Similarly, the analgesia quality in Group 5 was not better than those of the other groups in our study.

In their study, Stratmann et al. found lower VAS scores with a 5-mL volume and a 5-min lockout time [37]. However, that study was designed for labor analgesia. Unlike postoperative pain, labor pain is an increasing pain, and it is unstable. Moreover, the lockout time was set at 5 min, which was shorter than that in our study. For this reason, they may have had better analgesia in the 5 mL group. The side effects reported were similar to those in our study.

In a study conducted by Chen et al., levobupivacaine and fentanyl were used for PCEA after cesarean sections. The PCEA device settings were as follows: ‘bolus doses: 2 mL, lockout interval: 20 min, infusion dose: 3 mL/h’ [4]. Although these settings were lower when compared to our study in terms of the volume, they provided effective analgesia.

Halpern et al. reported that there were no ideal settings for the bolus dose and the lockout interval in PCEA, but better analgesia and maternal satisfaction could be provided with a high bolus dose of diluted anesthesia [31].

The results of our study showed that the initiation of PCEA with 20 mL of a diluted local anesthetic for postoperative analgesia in cesarean sections provided similar pain scores and less analgesic requirements, without a motor block and other side effects, during the early postoperative period when compared to the low volume initiations.

## Informed consent

This study was conducted after obtaining Ethics Committee approval from the İnönü University Medical Faculty (2015/171) and informed consent from all of the participants.
